# Estimating interactions and subgroup‐specific treatment effects in meta‐analysis without aggregation bias: A within‐trial framework

**DOI:** 10.1002/jrsm.1590

**Published:** 2022-07-28

**Authors:** Peter J. Godolphin, Ian R. White, Jayne F. Tierney, David J. Fisher

**Affiliations:** ^1^ MRC Clinical Trials Unit at UCL, Institute of Clinical Trials and Methodology University College London London UK

**Keywords:** covariate interaction, effect modifier, floating subgroup, meta‐analysis, subgroup analysis, within‐trial

## Abstract

Estimation of within‐trial interactions in meta‐analysis is crucial for reliable assessment of how treatment effects vary across participant subgroups. However, current methods have various limitations. Patients, clinicians and policy‐makers need reliable estimates of treatment effects within specific covariate subgroups, on relative and absolute scales, in order to target treatments appropriately—which estimation of an interaction effect does not in itself provide. Also, the focus has been on covariates with only two subgroups, and may exclude relevant data if only a single subgroup is reported. Therefore, in this article we further develop the “within‐trial” framework by providing practical methods to (1) estimate within‐trial interactions across two or more subgroups; (2) estimate subgroup‐specific (“floating”) treatment effects that are compatible with the within‐trial interactions and make maximum use of available data; and (3) clearly present this data using novel implementation of forest plots. We described the steps involved and apply the methods to two examples taken from previously published meta‐analyses, and demonstrate a straightforward implementation in Stata based upon existing code for multivariate meta‐analysis. We discuss how the within‐trial framework and plots can be utilised with aggregate (or “published”) source data, as well as with individual participant data, to effectively demonstrate how treatment effects differ across participant subgroups.

## 1 INTRODUCTION

Estimation of within‐trial interactions in meta‐analysis is considered crucial for reliable assessment of how treatment effects vary across participant subgroups.^1^ In recent years, a strong recommendation has emerged for a focus on covariate interactions derived within trials.^2^, ^3^ However, the within‐trial approach as previously described^2^ has various limitations. Where covariate interactions are identified, clinicians and policy makers ultimately need reliable estimates of effect specific to each patient subgroup—ideally expressed on an absolute scale—to allow treatments to be targeted at those who might benefit most and to inform patient choice. Furthermore, the existing method is unable to incorporate information from trials in which interactions cannot be estimated, such as those with all participants belonging to a single subgroup. Older approaches, whereby meta‐analyses are carried out separately for each subgroup and then compared, allow the straightforward estimation of such quantities and their presentation on forest plots. However, such approaches conflate within‐ and across‐trial information and are therefore prone to aggregation (or “ecological”) bias.^1^, ^4^, ^5^

In addition, while the described procedure for two subgroups was straightforward,^2^ it was not extended to the case of more than two subgroups, or for subgroups without a clear ordering or choice of reference group. Such cases require the estimation of more than one parameter, necessitating multivariate meta‐analysis. Importantly, this imposed unwanted restrictions on the range of patient covariates, potentially of clinical importance, that could be investigated in an appropriate way.

In this article, we extend our within‐trial interaction methodology^2^ to address these challenges. We intend these methods to be applied with observed effect sizes that are either extracted or calculated from aggregated source data,^6^, ^7^ although they may also be derived directly from individual participant data (IPD) as the first stage of a two‐stage analysis.^8^, ^9^ We provide a fully flexible framework to: (1) estimate within‐trial interactions across two or more subgroups, ordered or unordered, for categorical covariates; (2) estimate subgroup‐specific treatment effects (hereafter described as “floating” estimates) that make maximum use of available data and are compatible with the within‐trial interactions in the sense which we describe below; and (3) clearly present this data using novel implementations of forest plots.

The article is structured as follows: in Section 2 we describe motivating examples for this work, from two previously published meta‐analyses. We then proceed in Section 3 to describe the within‐trial framework and present the steps involved in estimating within‐trial interactions and compatible floating subgroup‐specific treatment effects. In Section 4 we apply these methods to data from our motivating examples. A discussion of the capabilities, further considerations and future developments of the framework is given in Section 5, and we finish with a brief conclusion in Section 6.

## 2 MOTIVATING EXAMPLES

Our motivating examples both come from published meta‐analyses in which detailed aggregate data were available, thus enabling us to apply the within‐trial framework.

### 2.1 Effects of interleukin‐6 antagonists on 28‐day all‐cause mortality in hospitalised COVID‐19 patients

Our first example is a collaborative, prospective meta‐analysis of the effects of interleukin‐6 antagonists, including tocilizumab, on outcomes for patients hospitalised with COVID‐19.^10^ One aim was to investigate whether there was a difference in the effect of tocilizumab on 28‐day all‐cause mortality in subgroups of patients who were and were not assigned to concomitant corticosteroids at baseline. A total of 15 trials were included: 1 that did not use corticosteroids, 2 that gave corticosteroids to all patients and 12 that administered corticosteroids to varying proportions of patients.

### 2.2 Effects of postoperative radiotherapy on survival of patients with non‐small cell lung cancer

Our second example is an IPD meta‐analysis which showed that post‐operative radiotherapy (PORT) was associated with poorer survival of patients with non‐small cell lung cancer. In the original analyses of these data,^11^, ^12^ using the older approach of pooling separately within subgroups, there was strong evidence that the negative effect of PORT increased with the number of affected lymph nodes. However, in the most recent update, the data were re‐analysed using a within‐trial approach, which showed much weaker evidence for such a trend.^13^ This meta‐analysis included 11 trials that provided data on patients' lymph node status, categorised as either *N*_0_, *N*_1_ or *N*_2_/*N*_3_. Only three of the trials recruited patients across all three lymph node categories, and four recruited patients only within one lymph node category. Although this was an IPD meta‐analysis, hazard ratios and confidence intervals were reported for each nodal status subgroup for each trial, allowing subgroup‐specific effect estimates and standard errors to be extracted.

## 3 GENERAL METHOD: THE WITHIN‐TRIAL FRAMEWORK

### 3.1 Model setup

Suppose we have *n* studies, within which patient observations are split into *k* disjoint subgroups. Let ${\hat{\beta }}_{\mathrm{ji}}$ represent the treatment effect estimated within subgroup *j* of trial *i*, and ${\hat{\beta }}_{i}$ be the vector of estimated subgroup‐specific treatment effects from trial *i*, with covariance matrix $S_{i}$. We suppose, at least for now, that the full set of subgroup‐specific treatment effects are observed for every trial, so there is no missing data. Then the standard multivariate meta‐analysis model is:

(1) $${\hat{\beta }}_{i}\sim \mathrm{MVN}\left(\beta ,S_{i}+\sum_{\beta }\right),\;$$

where ***β*** is the vector of subgroup‐specific treatment effects and $\sum_{\beta }$ is the between‐trial heterogeneity covariance matrix associated with ***β***, to be estimated from the data. Structures for $\sum_{\beta }$ are discussed in Section 3.2 below. Under this model alone, the standard estimator and variance gives results consistent with the older method of pooling within each subgroup separately.

Now, the interaction between treatment and the covariate from trial *i* may be represented by the *k* − 1 treatment effect contrasts ${\hat{\gamma }}_{i}$ with respect to a reference subgroup, with covariance matrix $V_{i}$. These effect vectors, which we refer to as “within‐trial interaction” vectors, are derived from ${\hat{\beta }}_{i}$ and $S_{i}$ via a simple linear combination, represented by the “contrast matrix” M. We may write another multivariate meta‐analysis model, similar to Equation (1), for the contrasts ${\hat{\gamma }}_{i}$:

(2) $$\begin{array}{c} {\hat{\gamma }}_{i}\sim \mathrm{MVN}\left(\gamma ,V_{i}+\sum_{\gamma }\right)\\ \text{where}\;{\hat{\gamma }}_{i}=M{\hat{\beta }}_{i},\;V_{i}=\mathrm{Var}\left({\hat{\gamma }}_{i}\right)=MS_{i}M^{T} \end{array}$$

Typically, covariances between the ${\hat{\beta }}_{\mathrm{ji}}$ at the trial level will be unreported but may be assumed to be negligible, so that $S_{i}$ is diagonal and we may write, where $J_{\left(k-1\right)}$ is the *k* − 1 by *k* − 1 matrix full of ones:

(3) $${\hat{\gamma }}_{i}=\left[\begin{array}{c} {\hat{\beta }}_{2i}-{\hat{\beta }}_{1i}\\ \vdots \\ {\hat{\beta }}_{\mathrm{ki}}-{\hat{\beta }}_{1i} \end{array}\right],\;V_{i}=S_{i1}J_{\left(k-1\right)}+\mathrm{diag}\left[S_{i2}\;\cdots \;S_{\mathrm{ik}}\right]$$

However, unless explicitly stated, nothing in what follows is affected by the precise form of $V_{i}$; the equations above demonstrate that non‐diagonal matrices $S_{i}$ are easily incorporated.

Under our proposed within‐trial framework, we wish for our subgroup‐specific estimates $\hat{\beta }$ to be compatible with the pooled within‐trial interactions $\hat{\gamma }$, in the sense that the contrasts of $\hat{\beta }$ are equal to $\hat{\gamma }$. Let $\hat{\theta }$ represent the subgroup‐specific treatment effect estimate in the reference subgroup, which without loss of generality we choose as *j* = 1. Then $\hat{\beta }$ is parameterised by $\hat{\theta }$ plus the *k* − 1 estimated contrasts $\hat{\gamma }$ with respect to that reference, under the relationships shown in Equation (4). In this scenario, we describe $\hat{\beta }$ as the “floating” subgroup‐specific effect estimates.

(4) $$\begin{array}{c} \hat{\beta }=1_{\left(k\right)}\hat{\theta }+\left[\begin{array}{c} 0\\ \hat{\gamma } \end{array}\right]=1_{\left(k\right)}\hat{\theta }+Z\hat{\gamma }\\ M\hat{\beta }=\hat{\gamma }\\ \hat{\mathrm{Var}}\left(\hat{\gamma }\right)=M\hat{\mathrm{Var}}\left(\hat{\beta }\right)M^{T},\;\sum_{\gamma }=M\sum_{\beta }M^{T} \end{array}$$

If, as stated above, the reference subgroup is identified by *j* = 1, then it is easily shown that $M=\left(-1_{\left(k-1\right)},I_{\left(k-1\right)}\right)$; that is, a column of length *k* − 1 full of ‘−1’ adjacent to the identity matrix of order *k* − 1. Similarly, we have the design matrix $Z={\left(0_{\left(k-1\right)},I_{\left(k-1\right)}\right)}^{T}$ acting on the contrast vector $\hat{\gamma }$. These two matrices have a simple and intuitive relationship (see Appendix 1.1) which holds regardless of the choice of reference factor.

### 3.2 Random effects considerations

We propose three basic forms for the heterogeneity covariance matrices $\sum_{\beta }$ and $\sum_{\gamma }$, described below in order of increasing complexity:

#### 3.2.1 Common‐effect

A fully common‐effect model—that is, with the interaction term(s) and the subgroup‐specific treatment effects common across studies—is obtained simply by setting both $\sum_{\gamma }$ and $\sum_{\beta }$ to zero.

#### 3.2.2 Exchangeable random‐effects

An exchangeable structure implies that heterogeneity variances and covariances do not depend on which subgroups are being compared. Given the constraint $\sum_{\gamma }=M\sum_{\beta }M^{T}$ (Equation 4), it can be shown that:

(5) $$\sum_{\gamma }=\frac{1}{2}\tau_{\gamma }^{2}\left(J_{\left(k-1\right)}+I_{\left(k-1\right)}\right),\;\sum_{\beta }=\left(\tau_{\beta }^{2}-\frac{1}{2}\tau_{\gamma }^{2}\right)J_{\left(k\right)}+\frac{1}{2}\tau_{\gamma }^{2}I_{\left(k\right)}$$

That is, we have a single heterogeneity parameter $\tau_{\gamma }^{2}$ shared across all estimated interaction contrasts; since contrasts are correlated due to their dependence on the reference subgroup, the correlation between each pair must be one‐half.^14^, ^15^ Similarly, we have a shared subgroup heterogeneity variance, $\tau_{\beta }^{2}$. Note that Equation (5) includes the special case where $\tau_{\gamma }^{2}$ is set to zero, implying $\sum_{\beta }=\tau_{\beta }^{2}J$, so that the contrasts γ are estimated under a common‐effect model with a shared heterogeneity variance $\tau_{\beta }^{2}$ both across and within subgroups.

#### 3.2.3 Unstructured random‐effects

Finally, we could allow $\sum_{\gamma }$ and $\sum_{\beta }$ to be unstructured, subject to the constraint $\sum_{\gamma }=M\sum_{\beta }M^{T}$ (Equation 4). This formulation allows a different heterogeneity variance to be estimated within each subgroup.

### 3.3 Estimation

In order to derive floating subgroup‐specific treatment effects, we first estimate the vector of pooled contrasts γ using a within‐trial approach. By subtracting this from the set of trial‐level subgroup effects ${\hat{\beta }}_{i}$, we proceed to estimate the treatment effect θ in the reference subgroup. Importantly, this ensures that all available data is used, and that the trial‐specific weighting is identical across subgroups (see Appendix 1.2). Finally, we associate the floating subgroup‐specific treatment effect in the reference subgroup $\beta_{1}$ with $\hat{\theta }$, and derive the remaining floating effects as shown in Equation (4). Hence, the floating subgroup‐specific effects are constrained to differ by the values of the pooled contrasts γ.

We present this approach below, broken down into three steps which are straightforward to implement. In general, the facility to estimate multivariate meta‐analysis models is required, such as “mvmeta” in Stata^14^, ^16^ or R^17^; sample Stata code to implement the framework under a common‐effect model is presented in Appendix 2, and a full Stata package “metafloat” is available via GitHub (https://github.com/UCL/metafloat). However, with a binary subgroup (*k* = 2) and under a common‐effect model, the approach simplifies considerably (see Appendix 1.5) such that the floating subgroup estimates and variances may be evaluated using spreadsheet software, or even by hand.

#### 3.3.1 Step 1. Estimate the within‐trial interaction(s)

Our first step is to apply the multivariate model for the interactions (Equation 2). In the general case, the standard estimator and variance under this model is:

(6) $$\hat{\gamma }={\left[\sum_{i=1}^{n}{\left(V_{i}+\sum_{\gamma }\right)}^{-1}\right]}^{-1}\sum_{i=1}^{n}{\left(V_{i}+\sum_{\gamma }\right)}^{-1}{\hat{\gamma }}_{i},\;\hat{\mathrm{Var}}\left(\hat{\gamma }\right)={\left[\sum_{i=1}^{n}{\left(V_{i}+\sum_{\gamma }\right)}^{-1}\right]}^{-1}$$

This is a straightforward generalisation of the existing concept of pooling within‐trial covariate interactions.^2^ The heterogeneity covariance matrix $\sum_{\gamma }$ may be estimated using restricted maximum likelihood (REML) either with an exchangeable structure or unstructured, as described in Section 3.2. A global test of interaction may be performed via a simultaneous Wald test of all elements of $\hat{\gamma }$ being equal to zero. With three or more subgroups in a natural ordering, it may be useful to perform an additional test for linear trend across the elements of $\hat{\gamma }$. This may be achieved with a simple modification to Equations (2) and (6); for further details see Appendix 1.4.

#### 3.3.2 Step 2. Estimate the floating subgroup‐specific treatment effects

We now subtract the fitted treatment effect contrasts $\hat{\gamma }$ from the observed subgroup‐specific treatment effects ${\hat{\beta }}_{2i}\ldots {\hat{\beta }}_{\mathrm{ki}}$ in the *non*‐reference subgroup(s) in each trial *i*. Together with the effects ${\hat{\beta }}_{1i}$ in the reference subgroup, we then have a set of values which we may interpret as estimates of the true underlying treatment effect θ in the reference subgroup. The standard estimator under the model described by Equations (1) and (2) is:

(7) $$\begin{array}{c} \hat{\theta }={\left[\sum_{i=1}^{n}1^{T}{\left(S_{i}+\sum_{\beta }\right)}^{-1}1\right]}^{-1}\sum_{i=1}^{n}1^{T}{\left(S_{i}+\sum_{\beta }\right)}^{-1}\left({\hat{\beta }}_{i}-Z\hat{\gamma }\right)\\ \hat{\mathrm{Var}}\left(\hat{\theta }\right)={\left[\sum_{i=1}^{n}1^{T}{\left(S_{i}+\sum_{\beta }\right)}^{-1}1\right]}^{-1} \end{array}$$

As stated in Section 3.1, the identification of the reference subgroup by *j* = 1 is arbitrary; see Appendix 1.3 for suggested modifications to the presented equations for a choice of reference other than the first subgroup. Again, the matrix $\sum_{\beta }$ may be estimated using REML; but now constraints are required on the values of certain matrix elements, dependent upon values taken from $\sum_{\gamma }$ (see Section 3.2; further details are given in Appendix 1.7). Having estimated $\hat{\theta }$, we form the vector of floating subgroup estimates $\hat{\beta }$ by adding to $\hat{\theta }$ the appropriate elements of $\hat{\gamma }$; see Equation (4). Note that at no point are different subgroup estimates compared between studies, so avoiding the risk of introducing aggregation bias.^2^

#### 3.3.3 Step 3. Correct the variances of the floating subgroup‐specific treatment effects

Although we have now obtained the point estimates for the floating effects, we have a little more work to do to obtain their variance. Observe that not only is $\hat{\beta }$ a random function of both $\hat{\theta }$ and $\hat{\gamma }$, but that $\hat{\theta }$ is itself a random function of $\hat{\gamma }$. Therefore, although Step 1 gives us $\mathrm{Var}\left(\hat{\gamma }\right)$, the apparent variance given by Step 2 only gives us $\mathrm{Var}\left(\hat{\theta }\left(\gamma \right)\right)$ evaluated at the fixed value $\gamma =\hat{\gamma }$. To resolve this, note from Equation (7) that $\hat{\theta }$ may be separated into two parts, only one of which is dependent on $\hat{\gamma }$. Therefore, with reference to Equation (4), we can express the vector of floating effects $\hat{\beta }$ as a linear combination of the ${\hat{\beta }}_{i}$ and $\hat{\gamma }$. As these are independent (see Appendix 1.1), the variance of $\hat{\beta }$ works out to take the following form:

(8) $$\mathrm{Var}\left(\hat{\beta }\right)=\hat{\mathrm{Var}}\left(\hat{\theta }\left(\gamma \right)\right)J_{\left(k\right)}+A\hat{\mathrm{Var}}\left(\hat{\gamma }\right)A^{T}$$

where the *k* by *k* − 1 matrix ***A*** is in terms of fixed, known quantities (see Appendix 1.1 for further details).

### 3.4 Accommodating studies where not all subgroups are observed

If any estimates ${\hat{\beta }}_{\mathrm{ji}}$ are unobserved for trial i, then ${\hat{\beta }}_{i}$ and ${\hat{\gamma }}_{i}$ contain estimates for just the observed subgroups. Similarly, the right‐hand side of Equations (1) and (2) contain the corresponding subvectors of β and γ, and submatrices of $S_{i}+\sum_{\beta }$ and of $V_{i}+\sum_{\gamma }$. This does not cause any technical issues for estimation. Rather, the unobserved estimates may be considered to be very imprecisely estimated, for example by assigning to them a value of zero for the effect size and a variance much exceeding the largest observed variance (e.g., in our examples below, a value of 10,000 was used). A similar approach has been suggested in related contexts^14^; we echo their recommendation to check that alternative values of the assigned variance give near‐identical results.

## 4 APPLICATION TO EXAMPLE DATA

We now demonstrate this methodology in practice using our two examples described in Section 2. The first example will primarily demonstrate the simplicity of calculations involved in the case of a binary covariate, whilst the second example demonstrates how to proceed in the case of covariate with three levels.

### 4.1 Effects of interleukin‐6 antagonists on 28‐day all‐cause mortality in hospitalised COVID‐19 patients

For this example, we use indices 1 and 2 to represent the “no corticosteroids” and “corticosteroids” groups respectively. The protocol [PROSPERO Identifier: CRD42021230155] specified a priori the use of a common‐effect model, which is used here in the first instance. As suggested in Section 2.2, to assist in calculation we assign a value of 0 to the unobserved subgroups for the effect size and 10,000 for the variance. Box 1 contains a detailed walk‐through of the within‐trial framework under a common‐effect model. Stata code to replicate this example is provided in Appendix 2.

BOX 1 **Demonstration of the three steps of the within‐trial framework under a common‐effect model using the interleukin‐6 antagonists meta‐analysis example**

**FIGURE 1:**
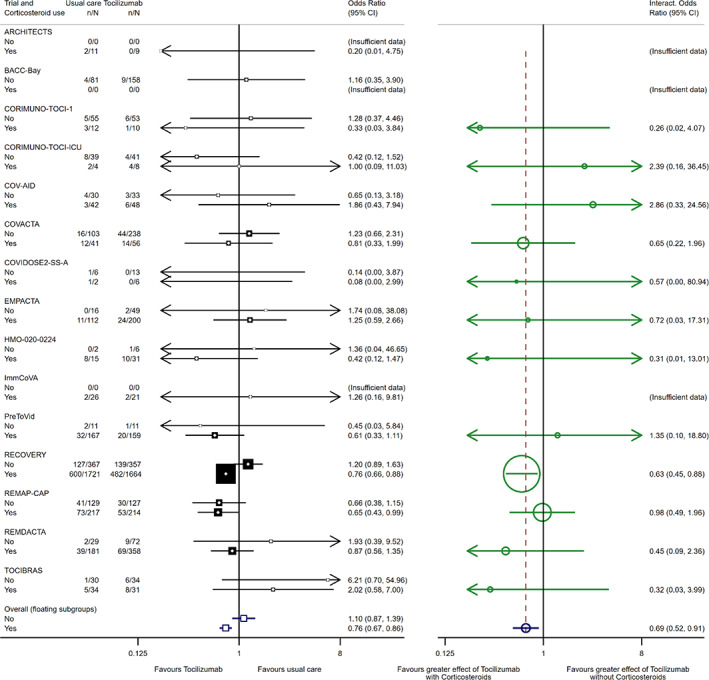
Interleukin‐6 antagonists meta‐analysis: Effects of tocilizumab on 28‐day all‐cause mortality by use of corticosteroids at baseline Each filled square denotes the odds ratio (OR) for each subgroup of patients defined by corticosteroid use at randomisation within each trial, with the horizontal lines showing the 95% CI. The dark blue open square represents the floating subgroup‐specific treatment effects for No corticosteroids and corticosteroids, with horizontal lines showing the 95% CI. Each green circle denotes the OR for the interaction between the effect of tocilizumab and corticosteroid use for each trial, with the horizontal lines showing the 95% CI. The dark blue circle represents a (common‐effect) meta‐analysis of the interaction ORs, with the horizontal line showing the 95% CI. [Colour figure can be viewed at wileyonlinelibrary.com]

**Step 1:** As we have a binary covariate and are using a common‐effect model, we may apply the simpler formulae given in Equations A12–A14 in Appendix 1.5. If No corticosteroids is chosen as the reference group, pooling the interactions (Equation A12) gives an estimate of $\hat{\gamma }=-0.370$ on the log odds ratio scale, with variance $\hat{\mathrm{Var}}\left(\hat{\gamma }\right)=0.020$. Exponentiating, we obtain $\exp\left(\hat{\gamma }\right)=0.69$ as shown in Figure 1.

**Step 2:** We next obtain estimates of the quantities $\hat{\mu }$, $A_{1}$ and $A_{2}$ (see Equation A13), and hence the floating subgroup effects ${\hat{\beta }}_{1}\left(=\hat{\theta }\right)$ and ${\hat{\beta }}_{2}$ constrained by the within‐trial interaction $\hat{\gamma }$. Our estimates are $\hat{\mu }=-0.189$, $A_{1}=-0.768$ and $A_{2}=A_{1}+1=0.232$; and hence we obtain ${\hat{\beta }}_{1}=\hat{\theta }=0.095$ and ${\hat{\beta }}_{2}=\hat{\theta }+\hat{\gamma }=-0.275$. Exponentiating to odds ratios, we have $\exp\left({\hat{\beta }}_{1}\right)=1.10$ and $\exp\left({\hat{\beta }}_{2}\right)=0.76$ as shown in Figure 1.

**Step 3:** In our final step, we refer to Equation A14. We find that $\mathrm{Var}\left(\hat{\mu }\right)=\mathrm{Var}\left(\hat{\theta }\right)=0.003$, and thence obtain $\mathrm{Var}\left({\hat{\beta }}_{1}\right)=0.003+0.020\times {\left(-0.768\right)}^{2}=0.015$ and $\mathrm{Var}\left({\hat{\beta }}_{2}\right)=0.003+0.020\times {\left(0.232\right)}^{2}=0.004$.

Indices 1 and 2 refer to "no corticosteroids" and "corticosteroids" groups respectively.

**FIGURE 2:**
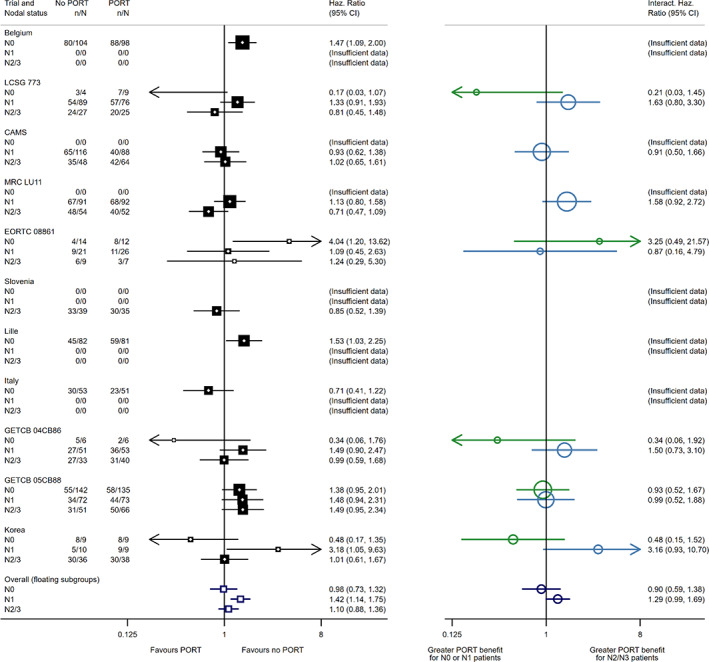
Post‐operative radiotherapy (PORT) for non‐small lung cancer meta‐analysis: Effect of PORT treatment on overall survival by nodal status. Green interactions represent the interaction between *N*_0_ and *N*_2/3_, light blue interactions represent the interaction between *N*_1_ and *N*_2/3_. Refer to Figure 1 for detailed descriptions on the various markers, except instead of odds ratios, these markers refer in the same way to hazard ratios. Note also that there are two pooled interaction hazard ratios represented by dark blue circles. The top dark blue open circle represents a (common‐effect) meta‐analysis of the interaction hazard ratios between *N*_0_ and *N*_2/3_, with the bottom dark blue open circle representing a (common‐effect) meta‐analysis of the interaction hazard ratios between *N*_1_ and *N*_2/3_. [Colour figure can be viewed at wileyonlinelibrary.com]

In Figure 1, we demonstrate presentation of the effect estimates for each subgroup within each trial on the left‐hand side, accompanied by the corresponding pooled floating subgroup‐specific treatment effects as derived above. The within‐trial interactions for each trial are shown on the right‐hand side of the plot, with the pooled interaction odds ratio (OR) of 0.69 (95% CI 0.52–0.91, *p* = 0.01), suggesting a greater benefit from Tocilizumab for patients that did receive corticosteroids compared to those who did not. Our floating subgroup‐specific treatment effect estimates for $\beta_{1}$ and $\beta_{2}$ translate to OR 1.10 (95% CI 0.87–1.39) and OR 0.76 (95% CI 0.67–0.86) respectively; note that their ratio exactly equals the pooled interaction estimate of 0.69. In this case, the results are similar to additional published results, which used the older method of pooling within each subgroup separately (Figure 2 of WHO REACT Group^10^), so here study conclusions remain unchanged. Supplementary random‐effects modelling showed no evidence of statistical heterogeneity in either interactions or subgroups ($\tau_{\beta }^{2}$ and $\tau_{\gamma }^{2}$ both <0.0001), and so results are not presented here.

### 4.2 Effects of postoperative radiotherapy on survival of patients with non‐small cell lung cancer

As described in Section 2.2, data from this IPD meta‐analysis had already been analysed using a within‐trial approach, assuming a linear trend across the three subgroups to estimate a single interaction.^13^ We now extend this example by estimating separate within‐trial interactions that compare effects both between participants with *N*_0_ (no affected lymph nodes) and *N*_1_ (one lymph node affected) disease to the reference group *N*_2/3_ (2 to 3 lymph nodes affected), and estimate floating subgroup‐specific treatment effects compatible with these interactions. We use the most up‐to‐date aggregate data, presented in the 2016 Cochrane review,^13^ rather than re‐analysing the IPD. We choose the *N*_2/3_ subgroup as the reference, as it is the best‐represented category among trials including more than one subgroup (see Appendix 1.3 for details of implementation when the reference is not the first subgroup). The floating subgroup‐specific treatment effects are presented in Figure 2, below the results for subgroups within each trial (as in Figure 1), and with within‐trial interactions with respect to the reference *N*_2/3_ subgroup on the right‐hand side.

Whereas the analysis using the older method of pooling within each subgroup separately suggested that PORT adversely affects the survival of participants with *N*_0_ disease, our floating estimate for *N*_0_ is near to null, similar to the *N*_2/3_ subgroup (see Supplementary Figure 1). Hence, where an across‐and‐within‐trial approach strongly suggests a trend (*χ* ^2^ = 4.51 on 1 d.f., *p* = 0.034), there is no such suggestion under our within‐trial framework (*χ* ^2^ = 0.31 on 1 d.f, *p* = 0.58). This difference is likely due to the Lille^18^ and Belgium^19^ trials, both of which included only *N*_0_ patients and reported large detrimental treatment effects. Under the older method, these trials heavily and directly influence the “naïve” pooled subgroup estimates; whereas under the within‐trial framework their influence is more indirect, as they do not contribute to the pooled interactions.

**FIGURE 3:**
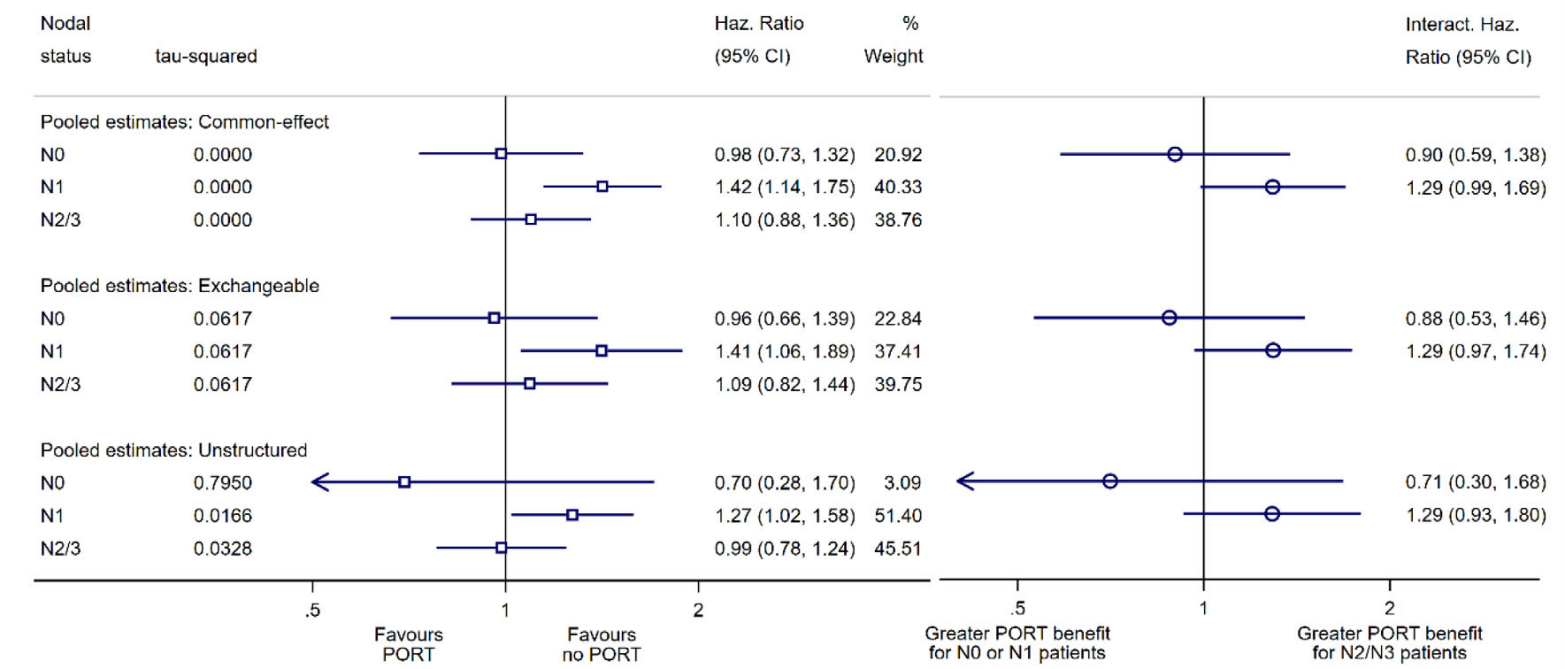
Post‐operative radiotherapy (PORT) for non‐small lung cancer meta‐analysis: Effect of PORT treatment on overall survival by nodal status. Results from a common‐effect model, and from random‐effects models with exchangeable and unstructured heterogeneity covariances Floating subgroup‐specific treatment effects (left panel) and pooled within‐trial interactions (right panel). The top dark blue open circle represents a meta‐analysis of the interaction hazard ratios between *N*_0_ and *N*_2/3_, with the bottom dark blue open circle representing a meta‐analysis of the interaction hazard ratios between *N*_1_ and *N*_2/3_. Note only summary information is presented on this plot and not trial‐level information as in Figures 1 and 2. [Colour figure can be viewed at wileyonlinelibrary.com]

Although common‐effect modelling was specified in the PORT protocol (available on request), we fit random‐effects models here as a demonstrative example (see Figure 3). Fitting an exchangeable structure results in a shared subgroup heterogeneity variance of $\tau^{2}=0.062$, such that the floating estimates all have moderately wider (compared to common‐effect) confidence intervals, whilst their point estimates remain roughly the same. However, fitting an unstructured model shows that there is substantially more heterogeneity within the *N*_0_ subgroup ($\tau^{2}=0.795$) than in the other two subgroups ($\tau^{2}=0.017$ and $\tau^{2}=0.033$ for *N*_1_ and *N*_2/3_ respectively). Hence, the *N*_0_ subgroup is now estimated noticeably differently, with an extremely wide confidence interval and with a point estimate shifted to the left (as a result of extreme estimates from small trials which are now given relatively greater weight). As a result, the floating estimates for *N*_1_ and *N*_2/3_ are also shifted moderately to the left, although in this particular example this does not affect the overall interpretation.

## 5 DISCUSSION

In this article, we extend the previously recommended^2^ within‐trial approach to a simple yet flexible framework for estimating treatment‐covariate interactions in meta‐analysis. Both ordered and unordered categorical covariates may be analysed, and heterogeneity covariance is handled straightforwardly. Crucially, we also provide practical methods of estimating floating treatment effects for each level of a covariate.

Previous criticism of testing for interactions using a within‐trial approach has included the potential for loss of power relative to older methods, for example from exclusion of “single‐subgroup” studies. By contrast, the within‐trial framework discussed here allows the entirety of information from all trials to be incorporated, whilst also taking account of the magnitude, direction and precision of the within‐trial pooled interaction. The floating subgroup‐specific treatment effects from this framework are compatible with within‐trial interactions—that is, each contrast is equal in magnitude to that of the corresponding interaction—and each is estimated using the same set of trial‐specific weights which reflect the totality of information in each trial. By comparison, pooling separately within subgroups assigns a different set of weights within each subgroup, making interpretation of subgroup contrasts difficult. Note that, when the subgroup of interest is fully observed and “balanced” in all trials (in the sense that $S_{i}=s_{i}I\;\forall i$), then subgroup‐specific treatment effects from our within‐trial framework and from the “pooling within subgroups” approach can be shown to coincide under a common‐effect model (see Appendix 1.6). Hence, we propose our framework as giving a more appropriate representation of comparative subgroup‐specific treatment effects. As such, where clinical decision‐making is best served by deriving additional statistics, such as translation (where appropriate) from relative to absolute scale,^20^, ^21^ this may be done most effectively via floating subgroup estimates.

### 5.1 Further considerations

We assume that treatment effects for the different subgroups within each trial can be extracted or calculated from aggregated source data,^6^, ^7^ or are derived directly from IPD. The results of a previous methodological review^2^ suggested that the availability of suitable aggregate data from peer‐reviewed articles was uncommon. However, this can be obtained through working collaboratively with trialists,^22^, ^23^ which allows prospective agreement on the important patient‐level covariates to analyse, as demonstrated in recent meta‐analyses in prostate cancer^24^, ^25^, ^26^ and COVID‐19.^10^, ^27^, ^28^

As discussed previously in both our examples, whilst studies including only a single participant subgroup cannot contribute to the within‐trial interaction, we can use their information in the within‐trial framework when estimating floating subgroup‐specific treatment effects compatible with this interaction. Doing so requires that we make the assumption of transitivity across subgroups: that is, that any non‐observed subgroup‐specific treatment effect could in principle have been observed, and that its true value (or its true distribution under a random‐effects model) would be identical to those of the remaining studies. If such studies (either singly, or taken together) are assigned relatively large weights, then this assumption may have a substantial impact upon the floating subgroup estimates. Conversely, if a “single‐subgroup” estimate is extreme relative to the remaining data, then it may be questionable whether the pooled interaction (and therefore the transitivity assumption) is applicable to that trial. In the context of our within‐trial framework, we therefore strongly recommend that reviewers critically evaluate the design and setting of “single‐subgroup” trials to assess whether the transitivity assumption holds, and whether construction of floating subgroup estimates including such trials would involve excessive extrapolation outside the range of observed data. Whilst this is not an issue for the examples presented in this paper, the illustrative example in Fisher et al.^2^ and the data presented in eFigure 1 of Sterne et al.^27^ are examples of situations where such evaluation might be recommended. One possible statistical approach here might be to include within the model described by Equation (4) an additional parameter, identifying “single‐subgroup” studies—or, more generally, the set of subgroups provided by each trial—analogously to identification of “designs” and inconsistency parameters in network meta‐analysis.^29^ As a sensitivity analysis, this approach might be used to test the effect of removing such studies from the estimation procedure.

The choice between common‐effect and random‐effects modelling is discussed elsewhere.^30^, ^31^ In the multivariate case, as here, the most general random‐effects model uses an unstructured covariance matrix, estimating a separate heterogeneity for each subgroup and thereby allows conclusions to be drawn regarding the source of heterogeneity or whether results from some subgroups are more reliably estimated than others. However, the number of parameters to be estimated increases sharply with the number of subgroups, and models might not converge with smaller datasets. Conversely, an exchangeable structure estimates a single heterogeneity parameter across all subgroups, with a common pairwise covariance. This simpler structure borrows strength across subgroups for the estimation of $\tau_{\beta }^{2}$. The special case of $\tau_{\gamma }^{2}=0$ places random‐effects on the subgroup estimates but not on the interaction estimates, and is simpler still. Note that we do not recommend random‐effects formulations which violate the compatibility relationships given in Equation (4).

A further consideration when utilising the within‐trial framework is presentation of the results on a forest plot. Where possible, we advocate presentation of the within‐trial interactions alongside the within‐trial subgroup‐specific treatment effects, as in Figures 1 and 2. However, for covariates with three levels or more and/or with large numbers of included studies (as in Figure 2), such plots become challenging to present clearly. Furthermore, although the choice of reference subgroup does not affect the pooled interactions or floating subgroup estimates themselves, it can have considerable impact on presentation. Specifically, if a trial does not contain the reference subgroup, then interactions cannot be plotted on the second panel of the plot for that trial. Typically, the reference subgroup should contain a relatively large amount of information; but it is possible that a suitable choice does not always exist. In such cases, we would recommend checking the “transitivity assumption” as described above; otherwise, displaying only the summary results (as in Figure 3 and Supplementary Figure 1), or only the left‐hand panel, may be a suitable alternative.

### 5.2 Future developments

The methods presented here may be generalised to encompass two‐stage IPD meta‐analysis^8^ which, among other things, would allow interactions with continuous covariates to be analysed in a within‐trial framework. However, our methods are not directly compatible with a one‐stage IPD meta‐analysis, in which a single generalised linear model is fitted to all trial data simultaneously. It has been noted^32^ that simply including a global treatment‐covariate interaction term into a one‐stage model would incorporate across‐trial information and is at risk of aggregation bias,^2^, ^3^ and so it has been recommended that within‐ and across‐trial information should be separated out to provide the equivalent one‐stage within‐trial interaction.^32^ Further possibilities to estimate patient‐level interactions exist for a one‐stage IPD meta‐analysis, with shrinkage methods suggested for use when a large number of treatment‐covariate interactions are to be modelled simultaneously.^33^ Further work is needed to extend the within‐trial framework into a one‐stage IPD meta‐analysis setting in order to estimate subgroup‐specific treatment effects that are compatible with patient‐level interactions and free from aggregation bias, and also in settings where “personalised” treatment effects utilising multiple covariates are desired.

## 6 CONCLUSIONS

Our within‐trial framework allows straightforward estimation of a range of within‐trial treatment‐covariate interactions, compatible subgroup‐specific treatment effects and corresponding forest plots, to clearly and effectively demonstrate how treatment effects differ across patient subgroups.

## AUTHOR CONTRIBUTIONS

Peter J. Godolphin: methodology; analysis and interpretation of data; writing ‐ original draft; writing ‐ review & editing. Ian R. White: conceptualization; methodology; software; writing ‐ review & editing. Jayne F. Tierney: conceptualization; acquisition of data; writing ‐ review & editing; project administration. David J. Fisher: methodology; software; analysis and interpretation of data; writing ‐ review & editing; supervision.

## FUNDING INFORMATION

David J. Fisher, Ian R. White and Jayne F. Tierney are supported by the UK Medical Research Council (https://mrc.ukri.org/) Grant number: MC_UU_00004/06. David J. Fisher and Peter J. Godolphin are part supported by Prostate Cancer UK (https://prostatecanceruk.org/) Grant number: RIA 16‐ST2‐020. Peter J. Godolphin is part supported by the National Institute for Health Research's Development and Skills Enhancement Award (NIHR301653). The funders had no role in study design, data collection and analysis, decision to publish, or preparation of the manuscript.

## CONFLICT OF INTEREST

The authors declare have no conflicts of interest.

## Supporting information


**Appendix S1** Supporting Information.

## Data Availability

All data used in this publication is aggregate data and is available within the paper. Stata code is also provided in Appendix 2 and is available via GitHub (https://github.com/UCL/metafloat).

## References

[1] Fisher DJ , Copas AJ , Tierney JF , Parmar MKB . A critical review of methods for the assessment of patient‐level interactions in individual patient data (IPD) meta‐analysis of randomised trials, and guidance for practitioners. J Clin Epidemiol. 2011;64:949‐967. 21411280 10.1016/j.jclinepi.2010.11.016

[2] Fisher DJ , Carpenter JR , Morris TP , Freeman SC , Tierney JF . Meta‐analytical methods to identify who benefits most from treatments: daft, deluded, or deft approach? BMJ. 2017;356:j573. 28258124 10.1136/bmj.j573 PMC5421441

[3] Riley RD , Debray TPA , Fisher D , et al. Individual participant data meta‐analysis to examine interactions between treatment effect and participant‐level covariates: statistical recommendations for conduct and planning. Stat Med. 2020;39(15):2115‐2137. 32350891 10.1002/sim.8516 PMC7401032

[4] Stewart GB , Altman DG , Askie LM , Duley L , Simmonds MC , Stewart LA . Statistical analysis of individual participant data meta‐analyses: a comparison of methods and recommendations for practice. PloS One. 2012;7(10):e46042. 23056232 10.1371/journal.pone.0046042 PMC3463584

[5] Berlin JA , Santanna J , Schmid CH , Szczech LA , Feldman HI . Anti‐lymphocyte antibody induction therapy study G. individual patient‐ versus group‐level data meta‐regressions for the investigation of treatment effect modifiers: ecological bias rears its ugly head. Stat Med. 2002;21(3):371‐387. 11813224 10.1002/sim.1023

[6] Tierney JF , Stewart LA , Ghersi D , Burdett S , Sydes MR . Practical methods for incorporating summary time‐to‐event data into meta‐analysis. Trials. 2007;8(1):16. 17555582 10.1186/1745-6215-8-16 PMC1920534

[7] Parmar MKB , Torri V , Stewart LA . Extracting summary statistics to perform meta‐analyses of the published literature for survival endpoints. Stat Med. 1998;17:2815‐2834. 9921604 10.1002/(sici)1097-0258(19981230)17:24<2815::aid-sim110>3.0.co;2-8

[8] Burke DL , Ensor J , Riley RD . Meta‐analysis using individual participant data: one‐stage and two‐stage approaches, and why they may differ. Stat Med. 2017;36(5):855‐875. 27747915 10.1002/sim.7141 PMC5297998

[9] Simmonds MC , Higgins JPT , Stewart LA , Tierney JF , Clarke MJ , Thompson SG . Meta‐analysis of individual patient data from randomised trials—a review of methods used in practice. Clin Trials. 2005;2(3):209‐217. 16279144 10.1191/1740774505cn087oa

[10] WHO Rapid Evidence Appraisal for COVID‐19 Therapies Working Group . Association between administration of IL‐6 antagonists and mortality among patients hospitalized for COVID‐19: a meta‐analysis. JAMA. 2021;326:499‐518. 34228774 10.1001/jama.2021.11330 PMC8261689

[11] PORT Meta‐analysis Trialists Group . Postoperative radiotherapy in non‐small‐cell lung cancer: systematic review and meta‐analysis of individual patient data from nine randomised controlled trials. Lancet. 1998;352:257‐263. 9690404

[12] Burdett S , Stewart L , PORT meta‐analysis group . Postoperative radiotherapy in non‐small cell lung cancer: an update of a systematic review and individual patient data meta‐analysis. Lung Cancer. 2005;49(2) S304:705. 10.1016/j.lungcan.2004.09.010 15603857

[13] Burdett S , Rydzewska L , Tierney J , et al. Postoperative radiotherapy for non‐small cell lung cancer. Cochrane Database of Syst Rev. 2016;10:CD002142. 27727451 10.1002/14651858.CD002142.pub4 PMC5642866

[14] White IR , Barrett JK , Jackson D , Higgins JPT . Consistency and inconsistency in network meta‐analysis: model estimation using multivariate meta‐regression. Res Synth Methods. 2012;3:111‐125. 26062085 10.1002/jrsm.1045 PMC4433771

[15] Salanti G , Higgins JPT , Ades AE , Ioannidis JPA . Evaluation of networks of randomized trials. Stat Methods Med Res. 2007;17(3):279‐301. 17925316 10.1177/0962280207080643

[16] White IR . Multivariate random‐effects meta‐regression: updates to mvmeta. Stata J. 2011;11(2):255‐270.

[17] Gasparrini A , Armstrong B , Kenward MG . Multivariate meta‐analysis for non‐linear and other multi‐parameter associations. Stat Med. 2012;31(29):3821‐3839. 22807043 10.1002/sim.5471 PMC3546395

[18] Lafitte JJ , Ribet ME , Prévost BM , Gosselin BH , Copin MC , Brichet AH . Postresection irradiation for T_2_ N_0_ M_0_ non‐small cell carcinoma: a prospective, randomized study. Ann Thorac Surg. 1996;62(3):830‐834. 8784014 10.1016/s0003-4975(96)00507-3

[19] van Houtte P , Rocmans P , Smets P , et al. Postoperative radiation therapy in lung cancer: a controlled trial after resection of curative design. Int J Radiat Oncol Biol Phys. 1980;6:983‐986. 6998936 10.1016/0360-3016(80)90105-4

[20] Tierney JF , Fisher DJ , Burdett S , Stewart LA , Parmar MKB . Comparison of aggregate and individual participant data approaches to meta‐analysis of randomised trials: an observational study. PLoS Med. 2020;17(1):e1003019. 32004320 10.1371/journal.pmed.1003019 PMC6993967

[21] Rothman KJ , Greenland S , Lash TL . Modern Epidemiology. Wolters Kluwer Health/Lippincott Williams & Wilkins; 2008.

[22] Tierney JF , Fisher DJ , Vale CL , et al. A framework for prospective, adaptive meta‐analysis (FAME) of aggregate data from randomised trials. PLoS Med. 2021;18(5):e1003629. 33956789 10.1371/journal.pmed.1003629 PMC8115774

[23] Godolphin PJ , Rogozińska E , Fisher DJ , Vale CL , Tierney JF . Meta‐analyses based on summary data can provide timely, thorough and reliable evidence: don't dismiss them yet. Nat Med. 2022;28:429‐430. 35145306 10.1038/s41591-021-01675-1

[24] Vale CL , Fisher D , Kneebone A , et al. Adjuvant or early salvage radiotherapy for the treatment of localised and locally advanced prostate cancer: a prospectively planned systematic review and meta‐analysis of aggregate data. Lancet. 2020;396:1422‐1431. 33002431 10.1016/S0140-6736(20)31952-8 PMC7611137

[25] Burdett S , Boeve LM , Ingleby FC , et al. Prostate radiotherapy for metastatic hormone‐sensitive prostate cancer: a STOPCAP systematic review and meta‐analysis. Eur Urol. 2019;76(1):115‐124. 30826218 10.1016/j.eururo.2019.02.003 PMC6575150

[26] Rydzewska LHM , Burdett S , Vale CL , et al. Adding abiraterone to androgen deprivation therapy in men with metastatic hormone‐sensitive prostate cancer: a systematic review and meta‐analysis. Eur J Cancer. 2017;84:88‐101. 28800492 10.1016/j.ejca.2017.07.003 PMC5630199

[27] WHO Rapid Evidence Appraisal for COVID‐19 Therapies Working Group , JAC S , Murthy S , et al. Association between administration of systemic corticosteroids and mortality among critically ill patients with COVID‐19: a meta‐analysis. JAMA. 2020;324(13):1330‐1341. 32876694 10.1001/jama.2020.17023 PMC7489434

[28] Godolphin PJ , Fisher DJ , Berry LR , et al. Association between tocilizumab, sarilumab and all‐cause mortality at 28 days in hospitalized patients with COVID‐19: a network meta‐analysis. PLoS One. 2022;17(7):e0270668. 35802687 10.1371/journal.pone.0270668 PMC9269978

[29] Higgins JPT , Jackson D , Barrett JK , Lu G , Ades AE , White IR . Consistency and inconsistency in network meta‐analysis: concepts and models for multi‐arm studies. Res Synth Methods. 2012;3:98‐110. 26062084 10.1002/jrsm.1044 PMC4433772

[30] Borenstein M , Hedges LV , Higgins JPT , Rothstein HR , Higgins DJPT . Introduction to Meta‐Analysis. John Wiley & Sons, Incorporated; 2011.

[31] Jackson D , Riley R , White IR . Multivariate meta‐analysis: potential and promise. Stat Med. 2011;30(20):2481‐2498. 21268052 10.1002/sim.4172 PMC3470931

[32] Hua H , Burke DL , Crowther MJ , Ensor J , Tudur Smith C , Riley RD . One‐stage individual participant data meta‐analysis models: estimation of treatment‐covariate interactions must avoid ecological bias by separating out within‐trial and across‐trial information. Stat Med. 2017;36(5):772‐789. 27910122 10.1002/sim.7171 PMC5299543

[33] Seo M , White IR , Furukawa TA , et al. Comparing methods for estimating patient‐specific treatment effects in individual patient data meta‐analysis. Stat Med. 2021;40(6):1553‐1573. 33368415 10.1002/sim.8859 PMC7898845

