# Supplementary material for: Estimating interactions and subgroup‐specific treatment effects in meta‐analysis without aggregation bias: A within‐trial framework
Source: Res Synth Methods. 2022 Jul 28;14(1):68–78. doi: 10.1002/jrsm.1590 (PMC10087172; doi:10.1002/jrsm.1590)
Supplement: Supplementary file 1 — Appendix S1 Supporting Information. [file JRSM-14-68-s001.docx]

# Appendices

## Appendix 1. Further statistical details

### Derivation of variance formula for floating subgroup-specific treatment effect estimates

As stated in the article body, our models for subgroup-specific treatment effects, and their contrasts with respect to a reference subgroup, are:

| ${\hat{\boldsymbol{\beta}}}_{\boldsymbol{i}}\sim MVN\left( \boldsymbol{\beta},\boldsymbol{S}_{\boldsymbol{i}}+\boldsymbol{\Sigma}_{\beta} \right), {\hat{\boldsymbol{\gamma}}}_{\boldsymbol{i}}\sim MVN\left( \boldsymbol{\gamma},\boldsymbol{V}_{i}+\boldsymbol{\Sigma}_{\gamma} \right)$  ${\hat{\boldsymbol{\gamma}}}_{i}=\boldsymbol{M}{\hat{\boldsymbol{\beta}}}_{i}, \boldsymbol{V}_{i}=\boldsymbol{M}\boldsymbol{S}_{\boldsymbol{i}}\boldsymbol{M}^{T}$ | (A1) |
| --- | --- |

where $\boldsymbol{M}=\left( -\boldsymbol{1}_{(k-1)},\boldsymbol{I}_{(k-1)} \right)$ is the contrast matrix.

Then, under our proposed within-trial framework, we have the following “consistency” relationships:

| $\boldsymbol{\beta=1}\theta\boldsymbol{+Z\gamma}$  $\boldsymbol{M\beta=M}\boldsymbol{1}\theta\boldsymbol{+MZ\gamma=0}\theta\boldsymbol{+I\gamma=\gamma}$  $Var\left( \boldsymbol{\gamma} \right)\boldsymbol{=M}Var\left( \boldsymbol{\beta} \right)\boldsymbol{M}^{T}, \boldsymbol{\Sigma}_{\gamma}=\boldsymbol{M}\boldsymbol{\Sigma}_{\beta}\boldsymbol{M}^{T}$ | (A2) |
| --- | --- |

where $\boldsymbol{Z=}\left( \boldsymbol{0}_{(k-1)},\boldsymbol{I}_{(k-1)} \right)^{T}$ is the covariate design matrix.

If we augment the matrix **M** above with the unit vector $\boldsymbol{e}_{1}$ with 1 in first position and zeroes elsewhere, and if we augment the matrix **Z** to the left with the constant term $\boldsymbol{1}_{\left( k \right)}$, then these two matrices are easily seen to be inverses of each other (Equation A3), as may be intuited from the expression for $\boldsymbol{\beta}$ given in Equation A2. This relationship holds regardless of the choice of reference subgroup.

| $\boldsymbol{M}^{*}=\left[ \begin{matrix} \boldsymbol{e}_{1} \\ \boldsymbol{M} \end{matrix} \right], \boldsymbol{Z}^{*}=\left[ \begin{matrix} \boldsymbol{1}_{\left( k \right)} & \boldsymbol{Z} \end{matrix} \right]$  $\boldsymbol{\beta=}\boldsymbol{Z}^{*}\left[ \begin{matrix} \theta\\ \boldsymbol{\gamma} \end{matrix} \right], \boldsymbol{M}^{*}\boldsymbol{\beta=}\left[ \begin{matrix} 0 \\ \boldsymbol{\gamma} \end{matrix} \right]\boldsymbol{,}\boldsymbol{M}^{*}=\left( \boldsymbol{Z}^{*} \right)^{-1}$ | (A3) |
| --- | --- |

Now, as described in the main text, Equation 8 allows us to split $\hat{\theta}$, and therefore also $\hat{\boldsymbol{\beta}}$, into two parts: one a random function only of ${\hat{\boldsymbol{\beta}}}_{i}$, and the other only of $\hat{\boldsymbol{\gamma}}$. To see this, we firstly define the quantity:

| $\hat{\mu}=\left[ \sum_{i=1}^{n} \boldsymbol{1}^{\boldsymbol{T}}\left( \boldsymbol{S}_{i}+\boldsymbol{\Sigma}_{\beta} \right)^{-1}\boldsymbol{1} \right]^{-1}\sum_{i=1}^{n} \boldsymbol{1}^{\boldsymbol{T}}\left( \boldsymbol{S}_{i}+\boldsymbol{\Sigma}_{\beta} \right)^{-1}{\hat{\boldsymbol{\beta}}}_{i}$ | (A4) |
| --- | --- |

which is interpretable as an estimate of the overall pooled treatment effect, stratified by subgroup and study membership. As such, it is independent of the pooled contrast vector $\hat{\boldsymbol{\gamma}}$. Next, we define the following *k* by *k*-1 matrix in terms of fixed, known quantities:

| $\boldsymbol{A}={-\boldsymbol{1}\left[ \sum_{i} \boldsymbol{1}^{\boldsymbol{T}}\left( \boldsymbol{S}_{i}+\boldsymbol{\Sigma}_{\beta} \right)^{-1}\boldsymbol{1} \right]}^{-1}\sum_{i} \boldsymbol{1}^{\boldsymbol{T}}\left( \boldsymbol{S}_{i}+\boldsymbol{\Sigma}_{\beta} \right)^{-1}\boldsymbol{Z}+\boldsymbol{Z}$ | (A5) |
| --- | --- |

…so that, putting Equations A4 and A5 together, we can write:

| $\hat{\boldsymbol{\beta}}\boldsymbol{=1}\hat{\theta}\boldsymbol{+Z}\hat{\boldsymbol{\gamma}}\boldsymbol{=1}\hat{\mu}+\boldsymbol{A}\hat{\boldsymbol{\gamma}}$ | (A6) |
| --- | --- |

From Equation 7 in the main text, we have that $Var\left( \hat{\mu} \right)=Var\left( \hat{\theta} \right)$; and since $\hat{\mu}$ and $\hat{\boldsymbol{\gamma}}$ are independent, Equation 8 follows immediately.

### Proof that each floating subgroup estimate has the same trial-specific weights

From previous equations, and noting that ${\hat{\boldsymbol{\gamma}}}_{i}=\boldsymbol{M}{\hat{\boldsymbol{\beta}}}_{i}$, it can be seen that $\hat{\theta}$, $\hat{\mu}$, and therefore each element of $\hat{\boldsymbol{\beta}}$, are all weighted sums of the ${\hat{\boldsymbol{\beta}}}_{i}$. We wish to find the trial-specific weighting matrices $\boldsymbol{W}_{i}$ such that:

| $\hat{\boldsymbol{\beta}}\boldsymbol{=}\sum_{i=1}^{n} \boldsymbol{W}_{i}{\hat{\boldsymbol{\beta}}}_{i}$ | (A7) |
| --- | --- |

We proceed by finding weighting matrices $\boldsymbol{W}_{i(1)}$ and $\boldsymbol{W}_{i(2)}$ for each of the two terms of Equation A6. Since this expression is linear, we may then simply sum these expressions to obtain $\boldsymbol{W}_{i}$. We find that:

| $\boldsymbol{W}_{i(1)}=\boldsymbol{1}\left[ \sum_{i=1}^{n} \boldsymbol{1}^{\boldsymbol{T}}\left( \boldsymbol{S}_{i}+\boldsymbol{\Sigma}_{\beta} \right)^{-1}\boldsymbol{1} \right]^{-1}\boldsymbol{1}^{\boldsymbol{T}}\left( \boldsymbol{S}_{i}+\boldsymbol{\Sigma}_{\beta} \right)^{-1}$  $\boldsymbol{W}_{i(2)}=\boldsymbol{A}\left[ \sum_{i=1}^{n} \left( \boldsymbol{V}_{i}+\boldsymbol{\Sigma}_{\gamma} \right)^{-1} \right]^{-1}\left( \boldsymbol{V}_{i}+\boldsymbol{\Sigma}_{\gamma} \right)^{-1}\boldsymbol{M}$  $\boldsymbol{W}_{i}=\boldsymbol{W}_{i(1)}+\boldsymbol{W}_{i(2)}$ | (A8) |
| --- | --- |

Now, the trial-specific weights associated with each *element* of $\hat{\boldsymbol{\beta}}$ are equal to the row totals of $\boldsymbol{W}_{i}$, which we may find via post-multiplication by $\boldsymbol{1}$. But note that $\boldsymbol{M}\boldsymbol{1=0}$, so that the contribution from $\boldsymbol{W}_{i(2)}$ disappears. Therefore, by applying some basic matrix algebra to the expression for $\boldsymbol{W}_{i(1)}$ above, we see that all floating subgroups are estimated using the same set of trial-specific weights, which are given by the following expression (which clearly totals one when summed over all trials *i*):

| $\left[ \sum_{i=1}^{n} \boldsymbol{1}^{\boldsymbol{T}}\left( \boldsymbol{S}_{i}+\boldsymbol{\Sigma}_{\beta} \right)^{-1}\boldsymbol{1} \right]^{-1}\boldsymbol{1}^{\boldsymbol{T}}\left( \boldsymbol{S}_{i}+\boldsymbol{\Sigma}_{\beta} \right)^{-1}\boldsymbol{1}$ | (A9) |
| --- | --- |

Equation A6 also allows us to interpret $\hat{\boldsymbol{\beta}}$ (and its variance) as “residuals” from the stratified overall pooled treatment effect $\hat{\mu}$. When the trial-specific weights are summed, the “residual” terms cancel out (expressed via $\boldsymbol{W}_{i(2)}$ and $\boldsymbol{M}\boldsymbol{1=0}$), leaving us with simply the weights associated with $\hat{\mu}$ (see Equation 7 in the main article, and recall that $Var\left( \hat{\mu} \right)=Var\left( \hat{\theta} \right)$).

### Choosing a reference subgroup other than the first subgroup

The above equations make the assumption, as also stated in the main article body, that the contrasts ${\hat{\boldsymbol{\gamma}}}_{i}$ (and therefore also $\boldsymbol{\gamma}$) are expressed with respect to a reference subgroup identifiable as the first element of ${\hat{\boldsymbol{\beta}}}_{i}$ (and therefore also of $\boldsymbol{\beta}$). If, instead, we choose a different subgroup to be the reference, then one approach is to apply an appropriate permutation matrix (denoted by $\boldsymbol{T}$, and otherwise equal to the identity matrix) to ${\hat{\boldsymbol{\beta}}}_{i}$ so that the reference is moved to first position; then proceed as above. Finally, we need to apply the inverse operation to the resulting vector to give the floating subgroups $\boldsymbol{\beta}$ their correct ordering. That is:

| ${\hat{\boldsymbol{\gamma}}}_{i}=\boldsymbol{MT}{\hat{\boldsymbol{\beta}}}_{i}, \boldsymbol{V}_{i}=Var\left( {\hat{\boldsymbol{\gamma}}}_{i} \right)\boldsymbol{=}\left( \boldsymbol{MT} \right)\boldsymbol{S}_{\boldsymbol{i}}\left( \boldsymbol{MT} \right)^{T}, \boldsymbol{\Sigma}_{\gamma}=\left( \boldsymbol{MT} \right)\boldsymbol{\Sigma}_{\beta}\left( \boldsymbol{MT} \right)^{T}$  $\hat{\theta}=\left[ \sum_{i=1}^{n} \boldsymbol{1}^{\boldsymbol{T}}\left( \boldsymbol{S}_{i}+\boldsymbol{\Sigma}_{\beta} \right)^{-1}\boldsymbol{1} \right]^{-1}\sum_{i=1}^{n} \boldsymbol{1}^{\boldsymbol{T}}\left( \boldsymbol{S}_{i}+\boldsymbol{\Sigma}_{\beta} \right)^{-1}\left( {\hat{\boldsymbol{\beta}}}_{i}-\boldsymbol{T}^{\boldsymbol{T}}\boldsymbol{Z}\hat{\boldsymbol{\gamma}} \right)$  $\boldsymbol{\beta=1}\theta\boldsymbol{+}\boldsymbol{T}^{\boldsymbol{T}}\boldsymbol{Z\gamma}$ | (A10) |
| --- | --- |

### Testing for trend

For three or more subgroups with a natural ordering, it may be desirable to test for trend, in addition to the standard global test of interaction (Equation 2 of the main article) . In particular, this is likely to offer greater power for testing for an interaction, since fewer degrees of freedom are required. Such a trend may be modelled by modifying Equation 2 as follows:

| $\hat{\gamma}_{i}\sim MVN\left( \boldsymbol{d}\gamma,\boldsymbol{V}_{i}+\boldsymbol{\Sigma}_{\gamma} \right)$ | (A11) |
| --- | --- |

Under random-effects, this implies a heterogeneity covariance structure of $\boldsymbol{\Sigma}_{\gamma}=\tau_{\gamma}^{2}\boldsymbol{d}\boldsymbol{d}^{T}$, from which it may be shown that $\boldsymbol{\Sigma}_{\beta}=\tau_{\beta}^{2}\boldsymbol{J}+\tau_{\gamma}^{2}\boldsymbol{Zd}\boldsymbol{d}^{T}\boldsymbol{Z}^{T}$. For a linear trend across three subgroups, we choose $\boldsymbol{d}={\begin{matrix} [1 & 2 \end{matrix}]}^{T}$; other (non-linear) constraints may be imposed in a similar way. The test for trend is then evaluated via a Wald test of $\hat{\gamma}=0$.

### Results for two subgroups under common-effects

With two subgroups, and under a common-effect model (that is, where $\boldsymbol{\Sigma}_{\beta}\boldsymbol{=}\boldsymbol{\Sigma}_{\gamma}=\boldsymbol{0}$), the floating subgroup effects and variances may be expressed simply in terms of the subgroup effects $\hat{\beta}_{1i}, \hat{\beta}_{2i}$ and their variances $S_{1i}, S_{2i}$.

Step 1: Estimate the pooled within-study interaction, by evaluating Equation 6 in the main text:

| $\hat{\gamma}=\left[ \sum_{i=1}^{n} \left( S_{1i}+S_{2i} \right)^{-1} \right]^{-1}\left[ \sum_{i=1}^{n} \left( S_{1i}+S_{2i} \right)^{-1}\left( \hat{\beta}_{2i}-\hat{\beta}_{1i} \right) \right], Var\left( \hat{\gamma} \right)=\left[ \sum_{i=1}^{n} \left( S_{1i}+S_{2i} \right)^{-1} \right]^{-1}$ | (A12) |
| --- | --- |

Step 2: Estimate the floating subgroup effects, by evaluating the quantities $\hat{\mu}$ and ***A*** as defined in Equations A4 and A5 above:

| $\hat{\mu}=\left[ \sum_{i=1}^{n} \left( {S_{1i}}^{-1}+{S_{2i}}^{-1} \right) \right]^{-1}\left[ \sum_{i=1}^{n} {S_{1i}}^{-1}\hat{\beta}_{1i}+{S_{2i}}^{-1}\hat{\beta}_{2i} \right]$  $A_{1}=-\left[ \sum_{i=1}^{n} \left( {S_{1i}}^{-1}+{S_{2i}}^{-1} \right) \right]^{-1}\left[ \sum_{i=1}^{n} {S_{2i}}^{-1} \right], A_{2}=A_{1}+1=\left[ \sum_{i=1}^{n} \left( {S_{1i}}^{-1}+{S_{2i}}^{-1} \right) \right]^{-1}\left[ \sum_{i=1}^{n} {S_{1i}}^{-1} \right]$  $\hat{\beta}_{1}=\hat{\mu}+A_{1}\hat{\gamma}=\hat{\theta}, \hat{\beta}_{2}=\hat{\mu}+A_{2}\hat{\gamma}=\hat{\mu}+\left( A_{1}+1 \right)\hat{\gamma}=\hat{\theta}+\hat{\gamma}$ | (A13) |
| --- | --- |

Step 3: Estimate the variances of the floating subgroup effects:

| $Var\left( \hat{\mu} \right)=\left[ \sum_{i=1}^{n} \left( {S_{1i}}^{-1}+{S_{2i}}^{-1} \right) \right]^{-1}$  $Var\left( \hat{\beta}_{1} \right)=Var\left( \hat{\mu} \right)+{A_{1}}^{2} Var\left( \hat{\gamma} \right)$  $Var\left( \hat{\beta}_{2} \right)=Var\left( \hat{\mu} \right)+{A_{2}}^{2} Var\left( \hat{\gamma} \right)=Var\left( \hat{\mu} \right)+\left( A_{1}+1 \right)^{2} Var\left( \hat{\gamma} \right)$ | (A14) |
| --- | --- |

### Proof that “floating” and “naïve” subgroup estimates coincide, under the common-effects model, when subgroups are fully observed and balanced within studies

We have, in this scenario (where ***M*** and ***Z*** are as defined in Appendix 1.1), that:

| $\boldsymbol{S}_{i}=s_{i}\boldsymbol{I,}\boldsymbol{V}_{i}\boldsymbol{=}s_{i}\boldsymbol{M}\boldsymbol{M}^{T}=s_{i}\left( \boldsymbol{I+J} \right)$ | (A15) |
| --- | --- |

…so that:

| $\hat{\boldsymbol{\gamma}}=\left[ \sum_{i=1}^{n} {s_{i}}^{-1}\left( \boldsymbol{I+J} \right)^{-1} \right]^{-1}\sum_{i=1}^{n} {s_{i}}^{-1}\left( \boldsymbol{I+J} \right)^{-1}{\hat{\boldsymbol{\gamma}}}_{i}=\left[ \sum_{i=1}^{n} {s_{i}}^{-1} \right]^{-1}\sum_{i=1}^{n} {s_{i}}^{-1}\boldsymbol{M}{\hat{\boldsymbol{\beta}}}_{i}$  $\boldsymbol{A}=-\frac{1}{k}\left[ \sum_{i=1}^{n} {s_{i}}^{-1} \right]^{-1}\left[ \sum_{i=1}^{n} {s_{i}}^{-1}\boldsymbol{J} \right]+\boldsymbol{Z=Z-}\frac{1}{k}\boldsymbol{J}$  $\boldsymbol{\Rightarrow A}\hat{\boldsymbol{\gamma}}=\left[ \sum_{i=1}^{n} {s_{i}}^{-1} \right]^{-1}\sum_{i=1}^{n} {s_{i}}^{-1}\left( \boldsymbol{Z-}\frac{1}{k}\boldsymbol{J} \right)\boldsymbol{M}{\hat{\boldsymbol{\beta}}}_{i}=\left[ \sum_{i=1}^{n} {s_{i}}^{-1} \right]^{-1}\sum_{i=1}^{n} {s_{i}}^{-1}\left( \boldsymbol{I-}\frac{1}{k}\boldsymbol{J} \right){\hat{\boldsymbol{\beta}}}_{i}$ | (A16) |
| --- | --- |

Now, note that:

| $\boldsymbol{1}\hat{\mu}=\frac{1}{k}\left[ \sum_{i=1}^{n} {s_{i}}^{-1} \right]^{-1}\sum_{i=1}^{n} {s_{i}}^{-1}\boldsymbol{J}{\hat{\boldsymbol{\beta}}}_{i}$ | (A17) |
| --- | --- |

…so that, when we form $\hat{\boldsymbol{\beta}}$ using Equation A6, the two terms in $\boldsymbol{J}{\hat{\boldsymbol{\beta}}}_{i}$ cancel, leaving us with:

| $\hat{\boldsymbol{\beta}}=\left[ \sum_{i=1}^{n} {s_{i}}^{-1} \right]^{-1}\sum_{i=1}^{n} {s_{i}}^{-1}{\hat{\boldsymbol{\beta}}}_{i}$ | (A18) |
| --- | --- |

Similarly, the expression for the covariance matrix of $\hat{\boldsymbol{\beta}}$**,** given by Equation 8 in the main text, may be evaluated in our present scenario as follows:

| $\frac{1}{k}\left[ \sum_{i=1}^{n} {s_{i}}^{-1} \right]^{-1}\boldsymbol{J}+\left[ \sum_{i=1}^{n} {s_{i}}^{-1} \right]^{-1} \left( \boldsymbol{Z-}\frac{1}{k}\boldsymbol{J} \right)^{\boldsymbol{T}}\left( \boldsymbol{I+J} \right)\left( \boldsymbol{Z-}\frac{1}{k}\boldsymbol{J} \right)\boldsymbol{=}\left[ \sum_{i=1}^{n} {s_{i}}^{-1} \right]^{-1}\left[ \frac{1}{k}\boldsymbol{J}+\boldsymbol{I}-\frac{1}{k}\boldsymbol{J} \right]$  $=\left[ \sum_{i=1}^{n} {s_{i}}^{-1} \right]^{-1}\boldsymbol{I}$ | (A19) |
| --- | --- |

It may be seen that, as desired, Equations A18 and A19 describe the naïve estimates and variances derived from simply pooling the subgroup-specific sets of observations $\hat{\beta}_{ji}$ independently across trials.

### Implementation of random-effects models

As stated in the main article body, individually Equations (6) and (7) are straightforward to estimate using standard multivariate meta-analysis routines. However, we additionally wish to constrain the heterogeneity covariate matrices such that $\boldsymbol{\Sigma}_{\gamma}=\boldsymbol{M}\boldsymbol{\Sigma}_{\beta}\boldsymbol{M}^{T}$. What follows is a description of how we have implemented this within the Stata environment; there may be alternative approaches.

We will find it convenient to work with a reparameterization of ${\hat{\boldsymbol{\beta}}}_{i}$ which includes (a) the set of contrasts with respect to the reference; and (b) the reference itself, which, for reasons which will become clear, we now choose to be the *final* subgroup, $\beta_{ki}$. Let this reparameterization be represented by the matrix $\boldsymbol{R}$:

| $\left[ \begin{matrix} \boldsymbol{\gamma}_{i} \\ \beta_{ki} \end{matrix} \right]=\boldsymbol{R}\boldsymbol{\beta}_{i}\boldsymbol{, R}=\left[ \begin{matrix} \boldsymbol{I}_{k-1} & -\boldsymbol{1} \\ \boldsymbol{0} & 1 \end{matrix} \right]$ | (A20) |
| --- | --- |

(As before, ultimately the reference may be chosen without loss of generality, via application of a suitable permutation matrix $\boldsymbol{T}$ prior to the reparameterization; see Appendix 1.2).

Our reparametrized model for the floating treatment effect $\theta$ in the reference subgroup is then:

| $\boldsymbol{R}\left( \boldsymbol{\beta}_{i}-\boldsymbol{Z}\hat{\boldsymbol{\gamma}} \right)\sim MVN\left( \theta\boldsymbol{e}_{k},{\hat{\boldsymbol{U}}}_{i}+\boldsymbol{\Sigma}_{U} \right), \boldsymbol{e}_{k}\boldsymbol{=}\left[ \begin{matrix} 0 & \cdots& \begin{matrix} 0 & 1 \end{matrix} \end{matrix} \right]^{\boldsymbol{T}}$ | (A21) |
| --- | --- |

…where, by construction of $\boldsymbol{R}$, and for some as-yet unknown vector **a** and scalar *b*:

| ${\hat{\boldsymbol{U}}}_{i}=\boldsymbol{R}{\hat{\boldsymbol{S}}}_{i}\boldsymbol{R}^{T}\boldsymbol{=}\left[ \begin{matrix} {\hat{\boldsymbol{V}}}_{i} & -\hat{Var}\left( \beta_{ki} \right) \\ -\hat{Var}\left( \beta_{ki} \right) & \hat{Var}\left( \beta_{ki} \right) \end{matrix} \right], \boldsymbol{\Sigma}_{U}\boldsymbol{=R}\boldsymbol{\Sigma}_{\beta}\boldsymbol{R}^{T}\boldsymbol{=}\left[ \begin{matrix} \boldsymbol{\Sigma}_{\gamma} & \boldsymbol{a} \\ \boldsymbol{a}^{\boldsymbol{T}} & b \end{matrix} \right]$ | (A22) |
| --- | --- |

Therefore, if we fit the model above for $\theta$ *under the constraint* that the upper-left quadrant of $\boldsymbol{\Sigma}_{U}$ is equal to our previously-estimated $\boldsymbol{\Sigma}_{\gamma}$, then we can obtain $\boldsymbol{\Sigma}_{\beta}$ as follows:

| $\boldsymbol{\Sigma}_{\beta}=\boldsymbol{R}^{\boldsymbol{-1}}\boldsymbol{\Sigma}_{U}\boldsymbol{R}^{\boldsymbol{-T}}\boldsymbol{,}\boldsymbol{R}^{\boldsymbol{-1}}=\left[ \begin{matrix} \boldsymbol{I}_{k-1} & \boldsymbol{1} \\ \boldsymbol{0} & 1 \end{matrix} \right]$ | (A23) |
| --- | --- |

The constraints on $\boldsymbol{\Sigma}_{U}$ may be achieved in various ways, depending partly upon the choice of statistical software. With the user-written package **mvmeta** in Stata and R (see references 16 and 17 of main article), the heterogeneity covariance matrix is parameterised by the elements of its Cholesky decomposition. Using standard results for block matrices, and denoting the Cholesky factors of $\boldsymbol{\Sigma}_{U}$ and $\boldsymbol{\Sigma}_{\gamma}$ by $\boldsymbol{L}_{U}$ and $\boldsymbol{L}_{\gamma}$ respectively, it can be shown that (for some scalar *c*):

| $\boldsymbol{\Sigma}_{U}\boldsymbol{=}\left[ \begin{matrix} \boldsymbol{\Sigma}_{\gamma} & \boldsymbol{a} \\ \boldsymbol{a}^{\boldsymbol{T}} & b \end{matrix} \right]\boldsymbol{=}\boldsymbol{L}_{U}{\boldsymbol{L}_{U}}^{T}\boldsymbol{=}\left[ \begin{matrix} \boldsymbol{L}_{\gamma} & \boldsymbol{0} \\ \boldsymbol{a}^{\boldsymbol{T}}{\boldsymbol{L}_{\gamma}}^{-T} & c \end{matrix} \right]\left[ \begin{matrix} {\boldsymbol{L}_{\gamma}}^{T} & {\boldsymbol{L}_{\gamma}}^{-1}\boldsymbol{a} \\ \boldsymbol{0} & c \end{matrix} \right]$ | (A24) |
| --- | --- |

For an *unstructured* variance structure, it follows that we need simply to constrain the elements of the upper-left block of $\boldsymbol{L}_{U}$ to be equal to the corresponding elements of $\boldsymbol{L}_{\gamma}$, with the remaining elements freely estimated.

From the definition of the *exchangeable* structure (see Section 2.4) and of the matrix $\boldsymbol{R}$, we find that:

| $\boldsymbol{\Sigma}_{U}=\boldsymbol{R}\boldsymbol{\Sigma}_{\beta}\boldsymbol{R}^{T}\boldsymbol{=}\left( \tau_{\beta}^{2}-\frac{1}{2}\tau_{\gamma}^{2} \right)\boldsymbol{RJ}\boldsymbol{R}^{T}+\frac{1}{2}\tau_{\gamma}^{2}\boldsymbol{R}\boldsymbol{R}^{T}\boldsymbol{=}\left[ \begin{matrix} \boldsymbol{\Sigma}_{\boldsymbol{\gamma}} & -\frac{1}{2}\tau_{\gamma}^{2}\boldsymbol{1} \\ -\frac{1}{2}\tau_{\gamma}^{2}\boldsymbol{1}^{T} & \tau_{\beta}^{2} \end{matrix} \right]\boldsymbol{,}\boldsymbol{\Sigma}_{\gamma}\boldsymbol{=}\frac{1}{2}\tau_{\gamma}^{2}\left( \boldsymbol{J+I} \right)$ | (A25) |
| --- | --- |

That is, the elements of $\boldsymbol{a}$ in Equation A24 also depend on $\tau_{\gamma}^{2}$, inducing an additional set of constraints on $\boldsymbol{L}_{U}$ in terms of ${\boldsymbol{L}_{\gamma}}^{-1}$ and $\tau_{\gamma}^{2}$; the bottom-right element $\tau_{\beta}^{2}$ alone is freely estimated.

## Appendix 2. Stata code for Example 1

*--------------------------------------------------------------------------*

// Stata code to reproduce Example 1 of Godolphin, White, Tierney, Fisher //

*--------------------------------------------------------------------------*

// Interleukin-6 antagonists meta-analysis (Shankar-Hari et al.)

// Effects of tocilizumab on 28-day mortality by use of corticosteroids at baseline

// NOTE: This code requires the following Stata commands from SSC:

* dataex (included with official Stata from v14.1 onwards)

* metan (latest version v4.06 July 2022)

// NOTE: Subgroup here is Corticosteroids (No/Yes)

// [CODE]

* Example generated by -dataex-. To install: ssc install dataex

clear

input str17 TrialName str3 Subgroup int(n0 e0 n1 e1)

"ARCHITECTS" "No" 0 0 1 0

"ARCHITECTS" "Yes" 11 2 9 0

"BACC-Bay" "No" 81 4 158 9

"BACC-Bay" "Yes" 1 0 3 0

"CORIMUNO-TOCI-1" "No" 55 5 53 6

"CORIMUNO-TOCI-1" "Yes" 12 3 10 1

"CORIMUNO-TOCI-ICU" "No" 39 8 41 4

"CORIMUNO-TOCI-ICU" "Yes" 4 2 8 4

"COV-AID" "No" 30 4 33 3

"COV-AID" "Yes" 42 3 48 6

"COVACTA" "No" 103 16 238 44

"COVACTA" "Yes" 41 12 56 14

"COVIDOSE2-SS-A" "No" 6 1 13 0

"COVIDOSE2-SS-A" "Yes" 2 1 6 0

"EMPACTA" "No" 16 0 49 2

"EMPACTA" "Yes" 112 11 200 24

"HMO-020-0224" "No" 2 0 6 1

"HMO-020-0224" "Yes" 15 8 31 10

"ImmCoVA" "No" 1 0 1 0

"ImmCoVA" "Yes" 26 2 21 2

"PreToVid" "No" 11 2 11 1

"PreToVid" "Yes" 167 32 159 20

"RECOVERY" "No" 367 127 357 139

"RECOVERY" "Yes" 1721 600 1664 482

"REMAP-CAP" "No" 129 41 127 30

"REMAP-CAP" "Yes" 217 73 214 53

"REMDACTA" "No" 29 2 72 9

"REMDACTA" "Yes" 181 39 358 69

"TOCIBRAS" "No" 30 1 34 6

"TOCIBRAS" "Yes" 34 5 31 8

end

// [/CODE]

*** Setup within-trial framework

gen long f1 = n1 - e1 // non-events in treatment arm

gen long f0 = n0 - e0 // non-events in control arm

metan e1 f1 e0 f0, or nogr study(Trial) by(Subgroup) nooverall iv

rename _ES y

gen double S = _seES^2

keep y S Trial Subgroup

// augment if missing subgroup data

replace S = 1e5 if missing(y)

replace y = 0 if missing(y)

// convert subgroup variable into "canonical" form, i.e. with consecutive values 1, 2, ...

label define subgroup_ 1 "No" 2 "Yes"

encode Subgroup, gen(subgroup) label(subgroup_)

order subgroup, after(Subgroup)

summarize subgroup

scalar k = r(max)

scalar k1 = k - 1

// convert study variable into "canonical" form, i.e. with consecutive values 1, 2, ...

egen study = group(Trial)

order study, after(Trial)

summarize study

scalar t = r(max)

*** Step 1: Estimate the within-trial interactions

// column vector of ones, of length k

mat ones = J(k, 1, 1)

// premultiplying by M means extracting the contrasts (see Appendix 1.1)

mat M = J(k1, 1, -1), I(k1)

// accumulate matrices for within-trial interactions

mat SinvSum = J(k1, k1, 0)

mat SinvySum = J(k1, 1, 0)

forvalues i = 1 / `=t' {

mkmat y if study==`i', matrix(b`i')

mkmat S if study==`i', matrix(W`i')

matrix W`i' = diag(W`i')

mat y`i' = M*b`i'

mat S`i' = M*W`i'*M'

mat SinvSum = SinvSum + invsym(S`i')

mat SinvySum = SinvySum + invsym(S`i')*y`i'

}

// estimate within-trial interactions

mat VarGammaHat = invsym(SinvSum)

mat GammaHat = VarGammaHat * SinvySum

*** Step 2: Estimate floating subgroup-specific treatment effects

// Covariate data matrix Z

mat Z = J(k, k1, 0)

mat Z[2, 1] = I(k1)

// estimate mu (see Appendix 1.1 and 1.2)

mat SinvRef = J(1, 1, 0)

mat SinvyRef = J(1, 1, 0)

forvalues i = 1 / `=t' {

mat SinvRef = SinvRef + ones'*invsym(W`i')*ones

mat SinvyRef = SinvyRef + ones'*invsym(W`i')*b`i'

}

mat VarMuHat = invsym(SinvRef)

mat MuHat = VarMuHat * SinvyRef

// estimate theta (see Equation 7 of main text)

mat SinvyRef = J(1, 1, 0)

forvalues i = 1 / `=t' {

mat SinvyRef = SinvyRef + ones'*invsym(W`i')*(b`i' - Z*GammaHat)

}

mat VarThetaHat = invsym(SinvRef)

mat ThetaHat = VarThetaHat * SinvyRef

// Note that "theta" is simply "mu" with an offset term equal to Z*GammaHat

// derive floating subgroup-specific effect sizes for all subgroups

mat BetaHat = ThetaHat[1,1]*ones + Z*GammaHat

*** Step 3: Estimate covariance matrix for floating effects

mat A = J(1, k1, 0)

forvalues i = 1 / `=t' {

mat A = A + ones'*invsym(W`i')*Z

}

mat A = Z - ones*VarThetaHat*A

// note that we could also, equivalently, have defined BetaHat as:

mat BetaHat = MuHat[1,1]*ones + A*GammaHat

// covariance matrix (Equation 8 in the main text)

mat VarBetaHat = VarThetaHat*J(k, k, 1) + A*VarGammaHat*A'

// construct matrix containing floating effects and variances, and display

mat floating = J(2, 2, 0)

mat floating[1, 1] = BetaHat'

mat floating[2, 1] = VarBetaHat[1,1]

mat floating[2, 2] = VarBetaHat[2,2]

mat colnames floating = No Yes

mat rownames floating = eff var_eff

mat list floating

******************************************************************

*** Alternative method, using simplified formulae in Appendix 1.2

// No matrix algebra is used, just simple summations

sort study subgroup

by study: gen double Gamma = y - y[_n-1] if _n==2

by study: gen double VarGamma = S + S[_n-1] if _n==2

gen double Vinv = 1 / VarGamma

gen double VinvGamma = Vinv * Gamma

summarize Vinv

scalar VarGammaHat = 1 / r(sum)

summarize VinvGamma

scalar GammaHat = r(sum) * VarGammaHat

gen double Sinv = 1 / S

gen double SinvMu = Sinv * y

gen double SinvTheta = Sinv * (y - (subgroup==2)*GammaHat)

// "subgroup==2" signifies the non-reference subgroup

// that is, only subtract GammaHat for observations in the non-reference subgroup

summarize Sinv

scalar VarThetaHat = 1 / r(sum)

summarize SinvTheta

scalar ThetaHat = r(sum) * VarThetaHat

summarize SinvMu

scalar MuHat = r(sum) * VarThetaHat

// sum the weights in non-reference subgroup, to form A1

summarize Sinv if subgroup==2

scalar A1 = -r(sum) * VarThetaHat

// sum the weights in reference subgroup, to form A2

// ... and show that A2 = A1 + 1

summarize Sinv if subgroup==1

scalar A2 = r(sum) * VarThetaHat

assert float(A2 - A1) == 1

// finally, calculate the floating subgroup effects and variances

// compare these with matrix "floating" defined above

scalar b1 = ThetaHat

scalar b2 = ThetaHat + GammaHat

// equations in terms of Mu instead of Theta

assert float(b1) == float(MuHat + A1*GammaHat)

assert float(b2) == float(MuHat + A2*GammaHat)

scalar V1 = VarThetaHat + (A1^2)*VarGammaHat

scalar V2 = VarThetaHat + (A2^2)*VarGammaHat

// display the values of b1, b2, V1 and V2

scalar list b1 b2 V1 V2

# Supplementary Figure

**Supplementary Figure 1:** Post-operative radiotherapy (PORT) for non-small lung cancer meta-analysis: Effect of PORT treatment on overall survival by nodal status. Analysis using an across- and within-trial approach and is at risk of aggregation bias. Pooled subgroup effects (left panel) and comparison on these (right panel)
